# Implementation of the London Measure of Unplanned Pregnancy in routine antenatal care: A mixed-methods evaluation in three London NHS Trusts

**DOI:** 10.18332/ejm/188118

**Published:** 2024-06-03

**Authors:** Jennifer A. Hall, Catherine Stewart, Bryony Stoneman, Tamsin Bicknell, Holly Lovell, Helen Duncan, Judith Stephenson, Geraldine Barrett

**Affiliations:** 1 Faculty of Population Health Sciences, Elizabeth Garrett Anderson Institute for Women's Health, School of Life and Medical Sciences, University College London, London, United Kingdom; 2 Women's Health Department, University College London Hospitals NHS Foundation Trust, London, United Kingdom; 3 Maternity Department, Homerton Healthcare NHS Foundation Trust, Homerton University Hospital, London, United Kingdom; 4 Women’s Health Department, Guy’s and St Thomas’ NHS Foundation Trust, St Thomas’ Hospital, London, United Kingdom; 5 Population Health Sciences Department, School of Life Course and Population Health, King’s College London, London, United Kingdom; 6 Department for Health and Social Care, Office for Health Improvement and Disparities, London, United Kingdom

**Keywords:** unplanned pregnancy, evaluation, implementation, London Measure of Unplanned Pregnancy, surveillance

## Abstract

**INTRODUCTION:**

Unplanned pregnancies are associated with increased risks. Despite this, they are currently not routinely detected during antenatal care. This study evaluates the implementation of the London Measure of Unplanned Pregnancy (LMUP) – a validated measure of pregnancy planning – into antenatal care at University College London Hospital, Homerton Hospital, and St Thomas’ Hospital, England, 2019–2023.

**METHODS:**

We conducted a mixed methods evaluation of the pilot. Uptake and acceptability were measured using anonymized data with non-completion of the LMUP as a proxy measure of acceptability overall. We conducted focus groups with midwives, and one-to-one interviews with women, to explore their thoughts of asking, or being asked the LMUP, which we analyzed with a Framework Analysis.

**RESULTS:**

Asking the LMUP at antenatal appointments is feasible and acceptable to women and midwives, and the LMUP performed as expected. Advantages of asking the LMUP, highlighted by participants, include providing additional support and personalizing care. Midwives’ concerns about judgment were unsubstantiated; women with unplanned pregnancies valued such discussions.

**CONCLUSIONS:**

These findings support the implementation of the LMUP in routine antenatal care and show how it can provide valuable insights into the circumstances of women's pregnancies. This can be used to help midwives personalize care, and potentially reduce adverse outcomes and subsequent unplanned pregnancy. Integration of the LMUP into the Maternity Services Data Set will establish national data collection of a validated measure of unplanned pregnancy and enable analysis of the prevalence, factors, and implications of unplanned pregnancies across subpopulations and over time to inform implementation.

## INTRODUCTION

Unplanned pregnancy is a crucial indicator to assess the success of sexual and reproductive health (SRH) programs and the fulfilment of the population’s SRH and rights^1^. It is associated with increased risks such as lower uptake of antenatal care, stillbirth, preterm birth, low birthweight, neonatal mortality, and postnatal depression^2-4^. Understanding the prevalence, distribution and impact of unplanned pregnancies is essential for addressing public health concerns, improving population health and maternity services, and reducing maternal mortality and morbidity^5^.

While the United States^6,7^ and many low- and middle-income countries have surveillance systems for unplanned pregnancies^8^, the United Kingdom relies on *ad hoc* surveys or proxy measures such as abortion data, which do not capture pregnancies that are continued to term. The most recent estimates for the UK are based on data from 2010–2012^9^. There is a need for up-to-date and ongoing surveillance of the prevalence and implications of unplanned pregnancies to inform policy development and maternity and reproductive health service planning.

The London Measure of Unplanned Pregnancy (LMUP) is a validated, concise tool that assesses the extent to which a recent or current pregnancy was planned^10-12^, on a scale from zero (most unplanned) to 12 (most planned). The LMUP, also known as the ‘Circumstances of Pregnancy’, questions are summarized below (full wording in Supplementary file S1):

1. Contraceptive use in the month of conception
2. The timing of becoming a mother (first time/again)
3. Pregnancy intention (before conception)
4. Desire for a baby (before conception)
5. Discussion of pregnancy with a partner (or decision to become pregnant alone)
6. Any preconception actions taken to prepare for pregnancy

The LMUP has been extensively researched across diverse populations^13-17^ and is recommended for use in the UK^18,19^ and the USA^20^, yet prior to this study it had not been implemented in routine care in England. The aim of this study is to describe the implementation and evaluation of the LMUP in antenatal care to inform national rollout.

## METHODS

A mixed-methods approach was taken comprising three steps: 1) analysis of anonymous antenatal data; 2) focus groups with midwives; and 3) one-to-one interviews with women who had completed the LMUP during antenatal care.

### Setting

The study was conducted in three maternity services in London, England: University College London Hospital (UCLH), Homerton Hospital (HH), and St Thomas’ Hospital (STH). In England women are encouraged to have their first antenatal appointment with a midwife, known as the ‘booking’ appointment, before the eleventh completed week of pregnancy. At ULCH and HH, the LMUP was included in the booking appointment whereas at STH the LMUP was available for self-completion by women as part of a pre-existing pre-booking workflow in the maternity app ‘Badgernotes’.

### Implementation

We sought approval from service leads and developed a technical specification for the changes required to the maternity information system (‘Medway’, then ‘EPIC’ at UCLH, a local build of ‘Cerner’ at HH, and ‘Badgernet’ at STH) which went through internal approval processes, commissioning, building and testing in 2017–2019 at UCLH, 2018–2019 at STH, and 2019–2020 at HH. In April 2019, UCLH changed to EPIC; however, problems with the EPIC rollout and the COVID-19 pandemic halted the pilot until April 2021. While the LMUP was available in Badgernet from early 2019, STH did not implement it until 2023. At HH the LMUP went live in August 2020 and was mandated, as is most of the booking appointment; at UCLH the LMUP was optional. UCLH and STH included the full wording of the LMUP whereas HH created a summarized version (Supplementary file S2).

At UCLH, JH met with midwives, facilitated by the Lead Midwife for Antenatal Care, and a midwife was employed 0.2WTE from May 2021 to support the implementation, including raising awareness via small group and 1:1 discussions, and evaluation at all sites. A video, covering the rationale and frequently asked questions, was sent to UCLH midwives and was included in the new staff pack, and posters were displayed in staff areas (UCLH) and via staff Instagram (HH) to maintain awareness. Specific training was not provided at HH or STH as locally it was felt that adaptation to using the LMUP would not be complex, especially as midwives at STH already asked whether the pregnancy was planned (yes/no), but staff were emailed about its introduction.

We worked with NHS Digital to create a space in the national Maternity Services Dataset^21^, and guidance and SNOMED codes for the LMUP^22^; LMUP data were successfully submitted and received by the Office for Health Improvement and Disparities from 2023.

### Evaluation

*Analysis of LMUP data*

Uptake and acceptability were measured using anonymized data from EPIC (UCLH) and Cerner (HH) from two months (August and September 2022) and from Badgernet (STH) for July–October 2023. This gave over 1000 women per site, considered an excellent sample size for evaluating a measure in new contexts^23^. Completion of the LMUP was used as a proxy for acceptability for UCLH and STH data where it was not mandatory. We calculated the Cronbach’s alpha (reliability) and confirmed unidimensionality with a Principal Components Analysis (structural validity). Data were analyzed using chi-squared tests to ascertain whether certain groups of women were less likely to complete the LMUP or certain questions, or whether midwives had concerns with any questions. For construct validity we tested our hypotheses, based on previous research^13-15,17^, that unplanned pregnancies would be more common in those aged <20 years, or who were unmarried/not in a relationship, or were parity three or higher. We used Kruskall-Wallis tests as LMUP score is non-parametric and because we were looking at differences across more than two groups.

*Focus groups with midwives*

The study was advertised to midwives via email. Focus groups were conducted with midwives who carry out booking appointments, according to a semi-structured topic guide (Supplementary file S3), to explore if/how they use the LMUP, what they liked or disliked about it, and how rollout of the LMUP could be improved. Given midwives at STH were not involved in asking the LMUP, we only conducted one focus group discussion (FGD) to explore their thoughts on the inclusion of the LMUP in the pre-booking workflow.

A mixture of eighteen community, clinic, and continuity midwives took part in three in-person FGDs at UCLH in February–June 2022. At HUH, six midwives took part in July–November 2022. The STH FGD with four midwives took place in May 2023. All FGDs and midwife interviews were facilitated by JH and either BS or CS, and took 40–55 min.

*One-to-one interviews with women*

The study was advertised to women through posters and flyers in booking packs. At STH, this was supplemented with targeted email invitations due to the low uptake of the pre-booking workflow (unrelated to the LMUP). We conducted one-to-one in-depth interviews with pregnant women who had had their booking appointment within the last four weeks (at point of contact) and had completed the LMUP questions (see Supplementary file S4 for topic guide). We planned to conduct up to 25 interviews.

Interviews and FGDs were conducted either in person at the Trust or online and were recorded using Zoom. All participants provided written informed consent. Field notes were made during interviews, and reflective notes afterwards, that were referred to during analysis. Video files were deleted once the transcription had been completed, checked and anonymized. Participants were asked if they wanted a copy of the transcript and none returned any comments. Transcribed interviews and FGDs were uploaded to Nvivo for analysis.

Recruitment took place at UCLH between February and May 2022. In all, 157 women expressed an interest, 31 were eligible, 20 were invited, and 13 were interviewed. At HH, recruitment took place July–October 2022; 24 women expressed an interest, 12 were eligible, 8 were invited, and 8 were interviewed. The most common reason for ineligibility was that their booking appointment was more than four weeks ago. We selected women from those who replied, to ensure we had a range of LMUP scores, ages, ethnicities and obstetric experiences, maximizing the information power of each interview^24^. At STH, low completion of the pre-booking workflow necessitated a more active recruitment strategy; however, limitations in the search function of the electronic maternity system meant that not all the potentially eligible women were identified. Consequently only 39 women were contacted within the study. Four were ineligible due to non-completion of the pre-booking workflow, four consented to interview and the remainder did not reply. Interviews were conducted by JH, BS or CS on Zoom, and lasted 30–40 minutes.

### Analysis

A thematic Framework Analysis^25-27^ aligned with the qualitive description approach^28^ was conducted to explore women’s and midwives’ thoughts about the LMUP; differences of opinion between women with unplanned compared to planned pregnancies were investigated.

JH developed a framework with high-level themes at the individual level of midwife and woman, organizational level and external factors, based on other research^29,30^, and sub-themes based on the data, and discussed with BS and CS. The transcripts were indexed by CS, BS and JH.

Participants are referred to with an identifier comprising their location, whether or not they were a midwife (MW), and a number. For example, HHMW24 refers to the setting of Homerton, MW means midwife, and 24 is the participant’s assigned number.

### Patient and public involvement

The patient and public involvement group aligned to the **P3 Study** (Pregnancy Planning, Preparation and Prevention), of which this research was part, were involved in discussions about the design and conduct of this research, reviewed participant documentation and topic guides, and discussed findings.

### Details of ethical approval

Ethical approval was granted by the South West, Cornwall and Plymouth Research Ethics Committee (REC reference: 21/SW/0174) for UCLH on 5 January 2022. The amendment to add HH was approved on 25 April 2022. The amendment to add STH was approved on 13 December 2022, and to send emails to potential participants was approved on 15 August 2023.

## RESULTS

### Analysis of anonymous antenatal data

Data on 1221 women were extracted from UCLH, 1110 from HH, and 1129 at STH. Population characteristics are shown in Table 1. The populations varied, reflecting the hospital’s catchment areas, with the highest average age at UCLH, greater ethnic diversity at STH, and more deprived populations at HH. Consequently, there was variation in the levels of pregnancy planning, with the highest proportion of planned pregnancies (LMUP score >9) at UCLH (84.2%) (Table 2). The LMUP performed as expected in terms of its psychometric properties at UCLH and STH; at HH there was a possible issue with question 1, but otherwise performed well (Supplementary file S5).

**Table 1:** Sociodemographic characteristics of women completing the London Measure of Unplanned Pregnancy in August–September 2022 at University College London Hospital and Homerton Hospital, and in July–October 2023 in St Thomas’ Hospital

*Characteristics*	*University College London Hospital (N=1221) n (%)*	*Homerton Hospital (N=1110) n (%)*	*St Thomas’ Hospital (N=1863) n (%)*
**Age** (years)			
Mean (SD), range	33.4 (5.30), 15–52	31.9 (5.73), 17–56	32.5 (5.4), 15–52
Median (IQR)	33 (18–48)	33 (18–46)	33 (17–48)
**Ethnicity**			
White	652 (53.4)	671 (60.5)	913 (49.0)
Black	113 (9.25)	163 (14.7)	349 (18.7)
Asian	144 (11.8)	134 (12.1)	199 (10.7)
Mixed	54 (4.42)	42 (3.78)	148 (7.9)
Other	134 (11.0)	88 (7.93)	186 (10.0)
Not stated	124 (10.16)	5 (0.45)	61 (3.27)
Missing	-	-	7 (0.38)
Relationship status*			
In a relationship	175 (14.3)	989 (89.1)	1694 (90.9)
Not in a relationship	69 (5.65)	121 (10.9)	79 (4.24)
Missing	977 (80.0)	-	90 (4.83)
**Index of Multiple Deprivation Decile**			
1	43 (3.52)	57 (5.14)	28 (1.5)
2	190 (15.6)	226 (20.4)	343 (18.4)
3	224 (18.4)	371 (33.4)	435 (23.4)
4	171 (14)	181 (16.3)	332 (17.8)
5	126 (10.3)	81 (7.3)	202 (10.8)
6	155 (12.7)	51 (4.59)	157 (8.43)
7	96 (7.86)	22 (1.98)	105 (5.64)
8	79 (6.47)	18 (1.62)	94 (5.05)
9	94 (7.7)	17 (1.53)	65 (3.49)
10	33 (2.7)	2 (0.18)	40 (2.15)
Missing	10 (0.82)	84 (7.57)	62 (3.33)
**Parity**			
Mean (SD), range	0.75 (1.19), 0–12	1.05 (1.72), 0–11	0.66 (0.96), 0–7
Median (IQR)	0 (0–7)	0 (0–10)	0 (0–6)
Missing	3 (0.25)	-	-
**Conceived with fertility treatment**			
Yes	124 (10.16)	NA	147 (7.89)
No	1049 (85.91)		1048 (56.3)
Missing	48 (3.93)		668 (35.9)

* Relationship status variable differed: at UCLH it was marital status, at HH it was whether the details field of the partner had been populated, and at STH it was whether they were in a relationship with the biological father. NA: not available. IQR: interquartile range.

**Table 2:** Uptake and completion of the London Measure of Unplanned Pregnancy in August–September 2022 at University College London Hospital and Homerton Hospital, and in July–October 2023 in St Thomas’ Hospital overall and by question, with prevalence of unplanned pregnancy by site

*LMUP*	*University College London Hospital ^a^ (N=1221)*	*Homerton Hospital ^b^ (N=1110)*	*St Thomas’ Hospital ^c^ (N=1863)*
	*n (%)*	*n (%)*	*n (%)*
**Completion status**			
Completed	893 (73.1)	1110 (100)	622 (33.4)
Partially completed	103 (8.44)	0	37 (1.98)
Not completed	225 (18.4)	0	1204 (64.6)
**Questions with missing data**			
Q1 - contraception	24 (2.41)	0	4 (0.61)
Q2 - timing	25 (2.51)	0	5 (0.76)
Q3 - intention	32 (3.21)	0	4 (0.61)
Q4 - desire	38 (3.82)	0	6 (0.91)
Q5 - partner	41 (4.12)	0	14 (2.13)
Q6 - preparation	35 (3.51)	0	10 (1.52)
**Pregnancy planning**			
Total, n	913*	1110	658*
LMUP range	0–12	1–12	1–12
LMUP median (IQR)	12 (1–12)	10 (1–12)	11 (2–12)
Planned pregnancies (LMUP score 10–12)	828 (84.2)	776 (69.9)	520 (79.0)
Ambivalent (LMUP score 4–9)	124 (14.9)	303 (27.3)	128 (19.5)
Unplanned pregnancies (LMUP score 0–3)	9 (0.92)	31 (2.79)	10 (1.52)

a Optional in booking.

b Mandatory in booking.

c Pre-booking self-completion.

* Where the LMUP was at least half complete the score was calculated using mean imputation, as recommended.

During the implementation at UCLH, completion of the LMUP increased from 51% in May 2021 to 85% in January 2022, largely due to the work of the LMUP champion. Completion of the LMUP questions by site is shown in Table 2; uptake shows clear differences according to the workflow. Partial completion was uncommon, especially at STH, and for each individual question there were <5% missing data at all sites, demonstrating acceptability. At UCLH, women with IVF pregnancies (n=124 vs n=1049) were less likely to be asked the LMUP (p<0.001) but there were no significant differences in completion by age, marital status or ethnicity. However, question five was more likely to be missing in women who were recorded as unmarried (n=69 vs n=175, p=0.015). Conversely, at STH, women aged <20 years (n=28 vs n=1832, p<0.001), who were not White or Asian (n=454 vs n=1402, p<0.001) or who were not in a relationship (n=79 vs n=1694, p<0.001) were less likely to complete the LMUP. The relationship with fertility treatment was more complex at STH, with the main difference being that women who conceived with fertility treatment were more likely to partially complete the LMUP (n=4/1048 vs n=9/147, p<0.001).

### Focus groups with midwives

The 18 midwives included seven clinic and 11 community or continuity midwives, ranging from newly qualified midwives to those with more than a decade’s experience. We did not collect data on their sociodemographic characteristics.

### One-to-one interviews with women

The characteristics of women are shown in Table 3. Most women had had at least one previous pregnancy (n=17, gravida 1–7), and some had experienced miscarriage, termination or IVF. Several had pre-existing medical conditions, including diabetes, cardiovascular disease and substance abuse. We interviewed women with a good spread of pregnancy intentions (LMUP score 2–12).

**Table 3:** Sociodemographic characteristics and London Measure of Unplanned Pregnancy score of women interviewed in February–May 2022 at University College London Hospital, July–October 22 at Homerton Hospital, and in July–December 2023 in St Thomas’ Hospital

*Participant code*	*Age (years)*	*Ethnicity*	*First pregnancy*	*Relationship*	*LMUP score*	LMUP category*
UCLH111	30–34	Black/Black British	No	Not given	9	Ambivalent
UCLH112	35–39	White	No	Yes	Incomplete (unplanned)	-
UCLH113	25–29	White	No	Not given	6	Ambivalent
UCLH114	35–39	Black/Black British	No	Yes	10	Planned
UCLH115	25–29	Asian/Asian British	No	Yes	12	Planned
UCLH116	35–39	Asian/Asian British	No	Yes	12	Planned
UCLH117	35–39	Black/Black British	Yes	Yes	6	Ambivalent
UCLH118	35–39	White	No	Yes	2	Unplanned
UCLH119	35–39	Chinese or Other	Yes	Yes	12	Planned
UCLH120	35–39	Black/Black British	No	Yes	12	Planned
UCLH121	30–34	White	Yes	Yes	12	Planned
UCLH122	30–34	White	No	Yes	Incomplete (planned)	-
UCLH123	25–29	White	Yes	Not given	5	Ambivalent
HUH124	30–34	Mixed	Yes	Yes	11	Planned
HUH125	35–39	White	No	Yes	10	Planned
HUH126	25–29	White	No	Yes	10	Planned
HUH127	30–34	White	Yes	Yes	12	Planned
HUH128	35–39	White	No	Yes	11	Planned
HUH129	35–39	Chinese or Other	No	Yes	6	Ambivalent
HUH130	35–39	White	No	Yes	8	Ambivalent
HUH131	25–29	Asian/Asian British	Yes	Yes	9	Ambivalent
STH132	30–34	White	Yes	Yes	7	Ambivalent
STH133	30–34	Asian/Asian British	No	Yes	12	Planned
STH135	30–34	Chinese or Other	No	Yes	12	Planned
STH136	35–39	White	No	Yes	10	Planned

* Planned (10–12), Ambivalent (4–9), Unplanned (0–3).

### Findings

**Figure 1:**
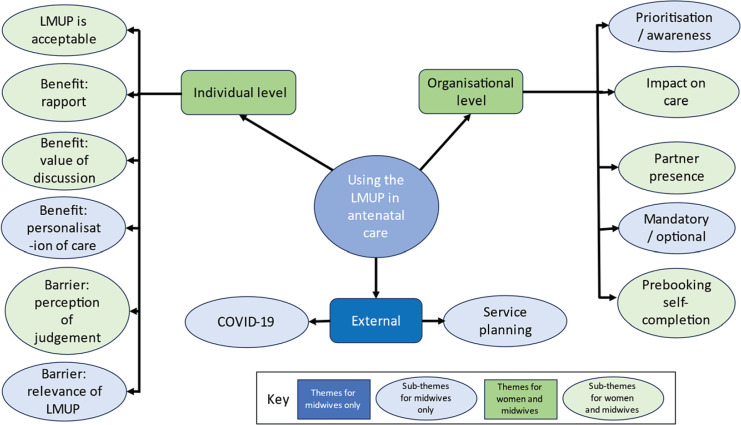
Themes and sub-themes identified within the overall framework of individual, organizational and external factors, from pregnant women and midwives interviewed in 2022 at University College London Hospital and Homerton Hospital, and in 2023 in St Thomas’ Hospital

The three themes and related sub-themes are shown in Figure 1. Given the overlap identified during analysis between what the women and midwives had said, some codes included midwives and women’s opinions together. Illustrative quotes are provided in Table 4.

**Table 4:** Illustrative quotes from pregnant women and midwives interviewed in 2022 at University College London Hospital and Homerton Hospital, and in 2023 in St Thomas’ Hospital

*Theme*	*Sub-theme*	*Illustrative quotes*
**Individual - midwife and/or woman**	**Attitude to LMUP**	*‘They tend to be quite easy questions to kind of ask.’,* (HHMW24, continuity midwife)
		*‘It’s nowhere near as personal as, you know, have social services been involved, the domestic violence ones, the have you been admitted as an inpatient to a psychiatric ward.’* (UCLHMW6, continuity midwife)
		*‘It felt like a flow of questions, it didn’t feel very intrusive or anything.’* (UCLH118, 35–39 years, LMUP score 2)
		*‘Yeah, I do remember her asking those questions, obviously they ask you quite a lot of personal stuff so it didn’t really stand out to me as being overly personal.’* (HH126, 25–29 years, LMUP score 10)
	**Benefits - rapport**	*‘I think it massively helps to know how to deal with the woman as an individual, and if you don’t ask the question, you could be leaving this massive gap that, you know, she walks out being like “oh that was nice but I’m still not feeling good [about the pregnancy]”.’* (UCLHMW11, continuity midwife)
		*‘I suppose actually you can help unpick … a person’s circumstances, if like there’s any issues in the household or, whatnot, it helps you divulge [sic] a little bit deeper and further into finding out about them and their needs.’* (UCLHMW18, continuity midwife)
		*‘I definitely felt like they were kind of with me when I was saying, oh, this was a bit of a surprise and it’s a bit difficult because blah, and they, they could, they were very kind of warm in their, umm hearing that and I felt like they understood what I was saying.’* (UCLHNW112, 35–39 years, LMUP incomplete - unplanned)
	**Benefits - value of discussion**	*‘I think it … gives you a little bit more of an insight as to what the situation is, and actually do we need to give them a little bit more support.’* (HHMW24, continuity midwife)
		*‘It’s quite nice having a professional there to process it with.’* (UCLH123, 25–29 years, LMUP score 5)
	**Benefits - personalization of care**	*‘… to look at whether, women are using contraception at the time when they fall pregnant or maybe in the lead up … and again, we can always look at it on the flip side after a woman has a baby, are we discussing contraception with her? Is she going home with contraception? And then, if not, are we finding her back a few months later?’* (HHMW23, community midwife)
	**Barriers - perception of judgement**	*‘It’s the women they are the most relevant for, they are going to feel the most targeted by them.’* (UCLHMW3, antenatal clinic midwife)
		*‘I did definitely feel like they understood my circumstances, … their attitude when I was answering the questions was very supportive and very, and they kind of chuckled along with it, when I was chuckling, or you know it felt very like, they were asking for the right reasons, like it was non-judgmental.’* (UCLH112, 35–39 years, LMUP incomplete - unplanned)
		*‘With most questionnaires, there’s always some random question, you know, like, you’ll go dentist, and they’ll ask “have you got HIV?” … So I wasn’t really shocked, or anything, or yeah, it didn’t feel like you were prying into, like you was asking too much personal questions or anything. I thought, I think it’s all relevant, especially where you’re going to be having a baby, you’re going to be giving life. It’s very important, anything that you can know about the mother and stuff, then I think it’s important, it should be noted.’* (STH132, 30–34 years, LMUP score 7)
	**Barriers - relevance of LMUP**	*‘Uhh, I don’t know if it’s relevant, because if you’re pregnant you’re pregnant it doesn’t really, I don’t really know if it matters how you get there.’* (UCLH121, 30–34 years, LMUP score 12)
		*‘Well, I think when you’re in a position where you’ve considered it really carefully and umm, and haven’t you know, you haven’t had to get over too many obstacles, it’s probably not as important as when it, as it would be if, if there were obstacles and there were issues. So, I fully understand where the questions are coming from and I think they’re really important.’* (UCLH122, 30–34 years, LMUP score incomplete - planned)
**Organizational**	**Prioritization**	*‘I didn’t understand why they were there, so I didn’t know what kind of importance there was, whereas I think you know learning about, this email coming out and this focus group, and I’m like oh that’s really important actually that we understand that information and we, we can refer on for people that maybe aren’t sure, and you know, give them those services … which makes me want to pay more attention to filling that box out accurately.’* (HHMW20, community midwife)
	**Impact of partner presence**	*‘Sometimes it kind of makes it easier, because sometimes the women totally forget stuff, and the partner tend to like speak up and like, answer the questions as well.’* (HHMW23, community midwife)
		*‘Mine wouldn’t [change], but I can imagine for some people they might have done.’* (HH128, 35–39 years, LMUP score 11)
	**LMUP being mandatory (HH only)**	*‘I do think though, if we didn’t have it as mandatory, hand on my heart, like the amount of times that you would just skip over it, because your time is short.’* (HHMW20, community midwife)
	**Self-complete pre-booking (STH only)**	*‘If you’re able to make a baby, you should be able to answer the questions by yourself … I think there’s some basic things the midwife shouldn’t have to take on honestly.* (STH132, 30–34 years, LMUP score 7)
		*‘It’s only a really small proportion of women that actually answer the pre booking questions before they actually come to their appointment. Usually nothing is completed, and you have to go through the whole booking from scratch at that appointment … when the ladies do fill out the question, it does make the appointment quicker … you just double check their answers with them and everything else.’* (STHMW27, community midwife)
**External**	**Benefits - service planning and public health surveillance**	*‘Still we’re having women that haven’t taken folic acid, haven’t taken vitamin D, so they’re not getting the information that they’re meant to have.’* (UCLHMW13, antenatal clinic midwife)
		*‘And I suppose, like, obviously from a public health point of view, like, to be looking at a national basis, I think more different ethnicities, different areas, … contraceptive services, particular age groups, that’s kind of what any demographic you know, is there anything that’s missing?’* (UCLHMW10, continuity midwife)

### Individual level

*LMUP is acceptable*

Across all settings, women found the LMUP easy to understand, and midwives found it easy to use. Women thought the questions fitted well within the booking appointment, whether in person or by self-completion in Badgernotes. Midwives agreed, saying that the addition of the questions had not made much difference to the booking appointment and that these questions were not as personal as others, such as those on domestic violence.

### Benefits

*Rapport*

The main benefit of asking the questions, according to midwives and women at UCLH and HH, was that the ensuing discussion can help build rapport and allow the midwife to get to know the woman, and her situation, better. This was one reason some of the women at STH thought that the questions would be best asked by a midwife rather than in the pre-booking, or at the very least should be discussed in the booking appointment.

*Value of discussion*

Midwives and women agreed that the questions could be used to open up valuable discussions not only about the circumstances of the pregnancy, but also about support, emotional wellbeing, relationships, continuing the pregnancy, safeguarding or general health. Women said that they thought these conversations would be especially valuable for women with unplanned pregnancies, with one woman saying that the questions led to a discussion that helped her process her unplanned pregnancy.

*Personalization of care*

Midwives thought that the questions were a good prompt for finding out health information about the woman, which can be used to identify any changes to care that need to be made or to trigger a conversation about health behaviors. It could be used to help identify women with other vulnerabilities, such as domestic violence or sexual abuse, or to prioritize discussions about postnatal contraception.

### Barriers

*Perception of judgement*

The main concern of midwives was the potential to make women feel judged or guilty, especially women with unplanned pregnancies and particularly with question 6 about taking action to prepare for the pregnancy. This was one reason why self-completion was suggested. By contrast, none of the women interviewed found the questions concerning, upsetting or judgmental. A few women, mainly those with planned pregnancies, thought that women in different circumstances might find the questions more sensitive. However, LMUP score was not associated with how women felt; women with unplanned pregnancies did not find the questions any less acceptable than women with planned pregnancies. Neither did women report feeling offended or guilty by question 6.

*Relevance of LMUP*

Some women were unsure about the relevance of the LMUP questions in general, suggesting that if you were pregnant, it did not matter how you got there. However, others could see why the questions were being asked, with some labeling them ‘important’, particularly if women had other challenges.

### Organizational level

*Prioritization and awareness*

At UCLH the main reason midwives did not ask the LMUP questions was time constraints; it was seen as a low priority due to the lack of associated action and poor knowledge about the link between unplanned pregnancy and adverse outcomes. At HH, midwives were unsure why the questions had been introduced and were unaware of the impact that unplanned pregnancies can have. Midwives generally agreed that if there was clear action triggered by the LMUP score and they better understood why the LMUP was being asked and the impact that unplanned pregnancies can have, then they would be more likely to ask the questions as intended.

*Impact on care*

Overall, most women felt that the LMUP questions had had little direct impact on their care, particularly those with the most planned pregnancies. When asked what services would be beneficial, women mentioned additional support, especially for those with unplanned pregnancies, in the form of counseling, group information sessions, face-toface appointments and signposting. Midwives at UCLH suggested that a specialized midwife providing support to women with low LMUP scores would be valuable, with midwives at HH suggesting women could be referred to their public health midwives. Other services mentioned were postnatal contraception, additional in-person antenatal appointments, early referral to their health visitor and additional postnatal care.

*Presence of partner*

All women said that their answers would not have changed if their partner was present. However, a few thought that for some other women, having their partner present might make it harder. Midwives were divided, with some finding it harder to ask or wondering if the woman might be less honest if their partner was present, while others said it was fine with the partners present, and that it can even be good as it involves the partner. At STH women are seen on their own for a few minutes at the start of booking, and both women and midwives suggested that this was a good opportunity for the LMUP questions.

*LMUP being mandatory*

Most HH midwives were happy with the LMUP questions being mandatory, like everything else in the booking. The only issue was regarding women who had experienced sexual abuse or rape, where an opt-out or free-text box was desired. Some UCLH midwives thought it should be made mandatory, but others preferred flexibility.

*Pre-booking self-completion*

Women and midwives at UCLH and HH independently suggested that women could complete the LMUP questions, and other information, before booking, saving time in the appointment. Some midwives thought women would be unlikely to do this, based on their previous experience. At STH, uptake of this workflow was low due to the number of steps women must take to download the app and log in. While the women we spoke to had successfully navigated this, most noted that it was a complex process. Once in the app, they found the whole questionnaire, LMUP included, straightforward. The benefits included being able to: complete it from the comfort of your own home at a convenient time; find out the answers (e.g. about a relative’s health) rather than being put on the spot; involving your partner should you wish; and relieving the burden on the midwife to free up time for more valuable conversations. Midwives at STH agreed that this content, and particularly family history, was well-suited to self-completion. On the rare occasion that the pre-booking information had been completed, it freed up time in the booking appointment as answers could be reviewed more quickly and the most important issues targeted.

### External level

*COVID-19*

Due to COVID-19, booking appointments were conducted by phone for parts of 2020 and 2021. When asked about the effect of this, midwives agreed that telephone bookings were harder than in-person appointments and they might have been more likely to skim over the LMUP questions.

*Service planning*

Midwives thought that the information gained from the LMUP questions could be used to plan preconception or contraception services more effectively, by highlighting, for example, the ongoing low levels of uptake of folic acid before booking.

### Suggestions

*Introducing the LMUP*

While most midwives start off the booking by alerting women to the fact that there are a lot of questions and everyone is asked the same, some thought that introducing the LMUP questions would help overcome concerns about sensitivity, judgement and relevance, and would allow women to answer more honestly. Some women at ULCH and HH also thought this would be beneficial. We developed, tested and agreed on the following introductory sentence in the July FGDs:

*‘The next questions ask about some of the circumstances around your pregnancy. We ask these to everyone, even though they may not always seem relevant. The purpose of these questions is to help us understand more about you and your pregnancy so that we can provide better care.’*

Women completing the questions in Badgernotes at STH generally did not feel the need for this.

## DISCUSSION

### Main findings

This is a novel study that evaluates the implementation of the LMUP in antenatal care in the UK. We have shown that it is feasible and acceptable to women and midwives in three large, busy London maternity services using different maternity information systems. Several advantages of asking the LMUP were highlighted by participants, including providing additional support and personalizing care, and concerns about judgment were not borne out; on the contrary women with unplanned pregnancies valued such discussions. Midwives highlighted an additional benefit that asking the LMUP questions can provide opportunities for women to disclose issues such as sexual abuse or substance misuse, though these were also reasons that sensitivity was needed. Disclosure can prompt discussions and referral to safeguarding, domestic abuse services, psychological support and other relevant support services and pathways. Women should be routinely asked about their mental health at each antenatal and postnatal visit. If a woman is flagged as having a lower LMUP score however, the importance of ensuring these enquiries are not missed could be emphasized, recognizing the higher prevalence of postnatal depression in this population.

Examination of the completion rate by question at UCLH showed that, where the LMUP was at least partially completed, question 6 (a list of actions taken prior to pregnancy) was the one that was most likely to be incomplete. This is in line with findings during the implementation about the challenges midwives experienced with this question. At UCLH each option (e.g. taking folic acid, achieving a healthy weight, stopping smoking) was listed separately with a yes/no tick box. Based on midwife feedback a ‘not applicable’ option was added. This was despite initial additional training and explanation that the focus of this question is on behavior change in preparation for pregnancy and not to assess the prevalence of behaviors. However, this did little to improve completion and we presented UCLH midwives with the summarized version used in HH and Sydney^29^, which has now been implemented at UCLH.

Other observed variation in completion at UCLH, including women with IVF pregnancies being less likely to be asked the LMUP and question 5 (partner) being more likely to be missing in unmarried women fits with what midwives said and is likely due to midwives not asking rather than women refusing. Interpretation of the differences seen in LMUP completion by sociodemographics is complicated by the different workflows. At UCLH it was reassuring to see that midwives were no more or less likely to ask the LMUP based on age, ethnicity, or marital status. The differences seen in STH on these variables may represent the varied uptake of the pre-booking workflow by women with these characteristics rather than completion of the LMUP itself, but we were unable to investigate this due to the limitations of the data.

### Interpretation

There is limited other evidence on the clinical implementation of the LMUP. An Australian evaluation of the use of the LMUP in antenatal care, which spoke only to midwives, found similar levels of general support and the same concerns regarding time constraints and the lack of associated action^29^. Analysis of LMUP completion in those two hospitals in Sydney, where the LMUP was asked by midwives and was not mandatory, showed significant differences between the sites, with 32.0% uptake at the tertiary referral hospital and 96.3% at the secondary hospital^31^. Important differences were noted, including that leadership support was less strong at the tertiary hospital, which resonates with our findings of importance of local leadership and LMUP champion. In addition, implementation coincided with COVID-19 and while bookings continued in-person at the secondary hospital, the tertiary hospital switched to telephone appointments^31^; this was noted in our study to reduce the likelihood that the LMUP was asked. At the tertiary hospital, LMUP completion rates were lower in women born overseas, whose preferred language was not English, or who had lower socioeconomic status; these were not factors considered in our study^31^.

A study of the potential introduction of the LMUP into early pregnancy units described clinician’s thoughts on asking about pregnancy intendedness as ‘polarized’, with some considering it essential and others insensitive^30^. Importantly, this study was hypothetical and, prior to being asked, none of the nurses interviewed knew of the LMUP. Surprisingly some thought that if the pregnancy was not continuing then it was not important to know, showing a lack of a holistic consideration of the woman’s needs; if that were an unplanned pregnancy a discussion of post-pregnancy contraception needs is indicated, in line with the Hatfield Vision recommendations^32^. Conversely women who were likely to try for another pregnancy would benefit from preconception advice.

It has been suggested that the LMUP is too complex to use, calculate or interpret in antenatal care^33^. This opinion, not based on an implementation or evaluation, is not upheld by our findings. Maternity information systems can be programmed to calculate and display the score to the midwife for action, as is the case now in HH where the LMUP score can trigger referrals regarding postnatal contraception; pathways that are being investigated elsewhere.

### Recommendations

*Local implementation*

- Share information around the time of the implementation on rationale, the relationships between unplanned pregnancy and adverse outcomes, how to ask the questions and what action to take in response, will support uptake.
- Training has a clear impact but it is hard to reach all midwives with face-to-face training. A combination of approaches (face-to-face, email, video, posters, one-to-one) is needed and needs to be regularly refreshed due to changes in the workforce. This can also be addressed by including learning about the use of the LMUP in new starter packs for midwives joining relevant areas.
- Having an LMUP champion, who can be a point of contact, answer questions and provide support during implementation of the LMUP is effective, as demonstrated by the high completion levels at UCLH where the LMUP is not mandatory. This role requires allocated time (2 hours per week during implementation periods) to maintain awareness until embedded.
- Listening to the feedback from the midwives and making changes where possible, without affecting the integrity of the measure, improves the engagement of the workforce.
- Linking the LMUP scores with actions will help to prioritize it; for example, at HH women with a low LMUP score (<4) are now considered for referral to public health midwifery and are flagged for additional support for postnatal contraception. Unplanned pregnancies are linked with other vulnerabilities, such as intimate partner violence and reproductive coercion^34, 35^. Each site should consider how the implementation of the LMUP can integrate into these existing pathways to provide women with unplanned pregnancies with the support that they need.

*National implementation*

- The inclusion of the LMUP questions in the Digital Maternity Record Standard^36^ will help overcome challenges and delays relating to the inclusion in maternity information systems.
- Using SNOMED codes, the LMUP data can be submitted to the Maternity Services Dataset^21^. Only complete LMUP scores should be submitted to MSDS, as incomplete scores being treated as complete leads to misclassification. The focus should be on achieving and maintaining high levels of completion.
- An NHS Futures platform has been developed on the Maternity Transformation Program’s workspace to showcase best practice and provide supporting materials for trusts to take on local implementation (https://future.nhs.uk/LocalTransformationHub/view?objectID=48872272).

*Future research*

- For a more complete picture implementation of unplanned pregnancy, the LMUP should be implemented and evaluated in Early Pregnancy Units and termination services, after appropriate consideration of the sensitivities of those settings.

### Strengths and limitations

A particular strength of this study is that we have incorporated the views of both those who collect (midwives) and provide (women) data at the booking appointment, and have triangulated this with data on completion for an in-depth evaluation. While our purposive sampling ensured as diverse a sample as possible, we were limited by who responded to advertisements. For example, no eligible women aged <26 years contacted the study. However, we were able to include women with unplanned pregnancies, the group midwives were most concerned about, and found that they valued the conversation the most. Evaluation in three sites has enabled a deeper learning of the barriers and facilitators to implementation by comparing the processes and findings. However, all three sites are in England where early antenatal care is led by midwives; consideration would need to be given to the transferability of these findings to settings where this is not the case. A limitation is that by only considering the booking appointment we are missing the women with the most unplanned pregnancies, who are seen in termination services or who may access antenatal care late or not at all, and those experiencing miscarriage, who may be seen in an early pregnancy unit. At STH it was not possible to identify women who had completed the pre-booking workflow but omitted the LMUP so we could not gain a complete picture of acceptability. The low level of completion mostly reflects the low uptake of the pre-booking workflow, which some midwives estimated to be as low as 10% in women attending antenatal clinics.

## CONCLUSIONS

This study has shown that the inclusion of the LMUP in antenatal care is acceptable to both women and midwives in a variety of workflows, paving the way for national implementation. The LMUP’s implementation in routine antenatal care can provide valuable insights into the circumstances of women’s pregnancies, help midwives personalize care, and potentially reduce adverse outcomes and subsequent unplanned pregnancy. By integrating LMUP data into the routine Maternity Services Data Set, it is possible to establish national data collection for a population-level measure of unplanned pregnancy in the Public Health Outcomes Framework. This framework, published as statutory guidance for local authorities, can serve as a key outcome measure to monitor progress in SRH goals, provide an endpoint by which to evaluate preconception interventions, and establish an ongoing public health surveillance system for unplanned pregnancies. This will contribute to our understanding of the prevalence, factors, and implications of unplanned pregnancies across different subpopulations and can inform strategies to improve reproductive and maternity healthcare, inequalities and outcomes.

## Supplementary Material



## Data Availability

The data supporting this research cannot be made available for privacy or other reasons. We do not have permission to share the data extracted from the health services. Anonymous transcripts of the qualitative data are available from the authors on reasonable request.
