# Supplementary material for: Implementation of the London Measure of Unplanned Pregnancy in routine antenatal care: A mixed-methods evaluation in three London NHS Trusts
Source: Eur J Midwifery. 2024 Jun 3;8:10.18332/ejm/188118. doi: 10.18332/ejm/188118 (PMC11145722; doi:10.18332/ejm/188118)
Supplement: Supplementary file 1 [file EJM-8-26-s1.pdf]

Supporting Information 1 – LMUP Questions and Scoring

Introduction

The next questions ask about some of the circumstances around your pregnancy. We ask these to everyone, even though they may not always seem relevant. The purpose of these questions is help us understand more about you and your pregnancy so that we can provide better care.

Questions 1 - 5

1. In the month that you became pregnant, would you say:
    - *a.* You were not using contraception *(score = 2)*
    - *b.* You were using contraception, but not on every occasion (like you sometimes used condoms or you missed some pills) *(score = 1)*
    - *c.* You always used contraception, but knew that the method had failed at least once (like the condom broke) *(score = 1)*
    - *d.* You always used contraception *(score = 0)*

2. In terms of becoming a mother (for the first time or again), do you feel that your pregnancy happened at the:
    - *a.* Right time *(score = 2)*
    - *b.* OK, but not quite right time *(score = 1)*
    - *c.* Wrong time *(score = 0)*

3. Just BEFORE you became pregnant would you say:
    - *a.* You intended to get pregnant *(score = 2)*
    - *b.* Your intentions kept changing *(score = 1)*
    - *c.* You did not intend to get pregnant *(score = 0)*

4. Just BEFORE you became pregnant would you say:
    - *a.* You wanted to have a baby *(score = 2)*
    - *b.* You had mixed feelings about having a baby *(score = 1)*
    - *c.* You did not want to have a baby *(score = 0)*

Thinking about your “partner”, if you have one, this might be (or have been) your husband/wife/civil partner, a partner you live with, a partner who lives elsewhere, someone you’ve had sex with once or twice, or a parenting (non-romantic) partner

5. BEFORE you became pregnant would you say:
    - *a.* You and your partner had agreed that you would become pregnant *(score = 2)*
    - *b.* You and your partner had discussed having children together, but hadn't agreed for you to become pregnant *(score = 1)*
    - c. You and your partner never discussed having children together *(score = 0)*
    - *d.* You chose to become pregnant without a partner *(score = 2)*

Options for Q6

Q6 can be written in one of the following ways:

BEFORE you became pregnant, did you do any of the following to improve your health in preparation for pregnancy? (Option to add a comment on each of the below)

- Did you take folic acid?
  - Yes
  - No
- Did you take vitamin D?
  - Yes
  - No
- Did you stop or cut down smoking?
  - Yes
  - No
  - N/A
- Did you stop or cut down drinking alcohol?
  - Yes
  - No
  - N/A
- Did you stop or cut down caffeine consumption?
  - Yes
  - No
  - N/A
- Did you eat more healthily?
  - Yes
  - No
- Did you achieve a healthy weight?
  - Yes
  - No
- Did you seek medical advice?
  - Yes
  - No
- Did you take some other actions? If yes, please comment.
  - Yes
  - No

2 options selected with "yes" gives a score of 2, if 1 "yes" option is selected then score is 1, if all answers are "no" or "N/A" then this will give a score of 0.

OR

BEFORE you became pregnant, did you do any of the following to improve your health in preparation for pregnancy? e.g., taking folic acid or vitamin D, reducing smoking, alcohol or caffeine, eating more healthily, achieving a healthy weight, seeking medical advice or any other action.

- 2 or more health preparation actions taken *(score = 2)*
- 1 health preparation action taken *(score = 1)*
- No health preparations *(score = 0)*

Supporting Information 2 – LMUP in EPIC (UCLH) and Cerner (HH)

UCLH

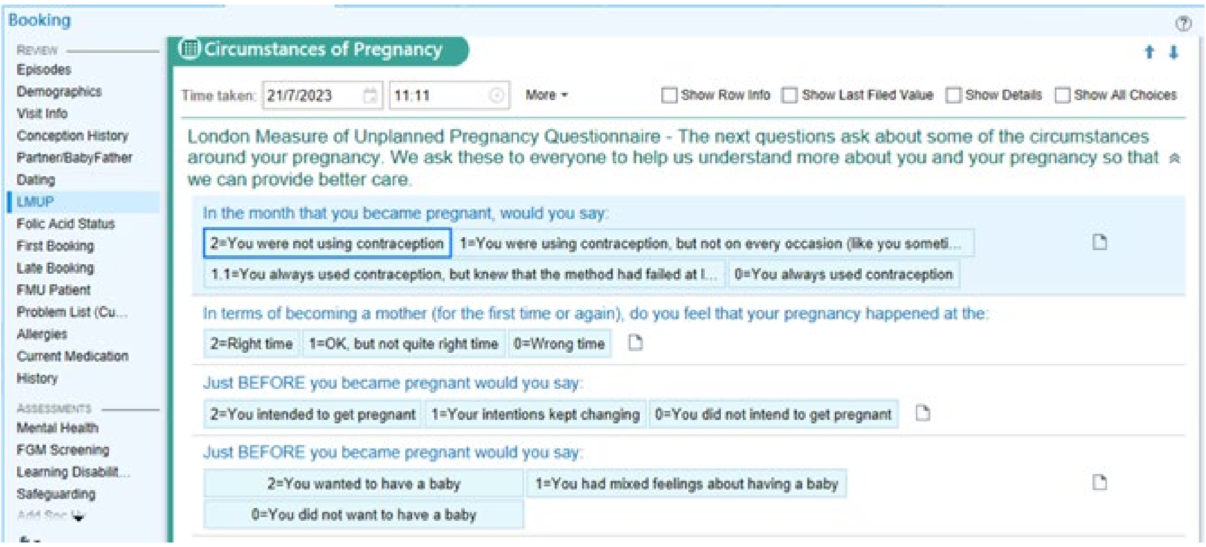

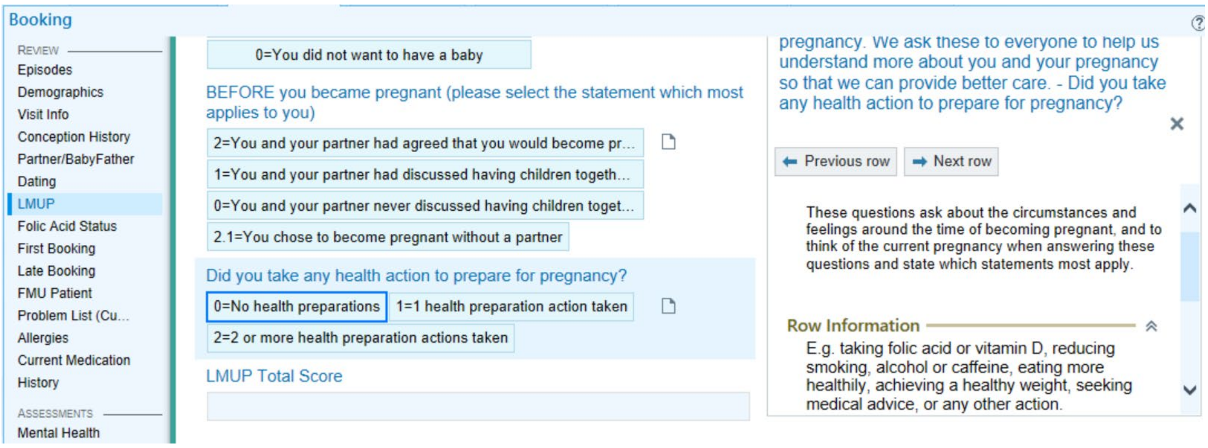

HH

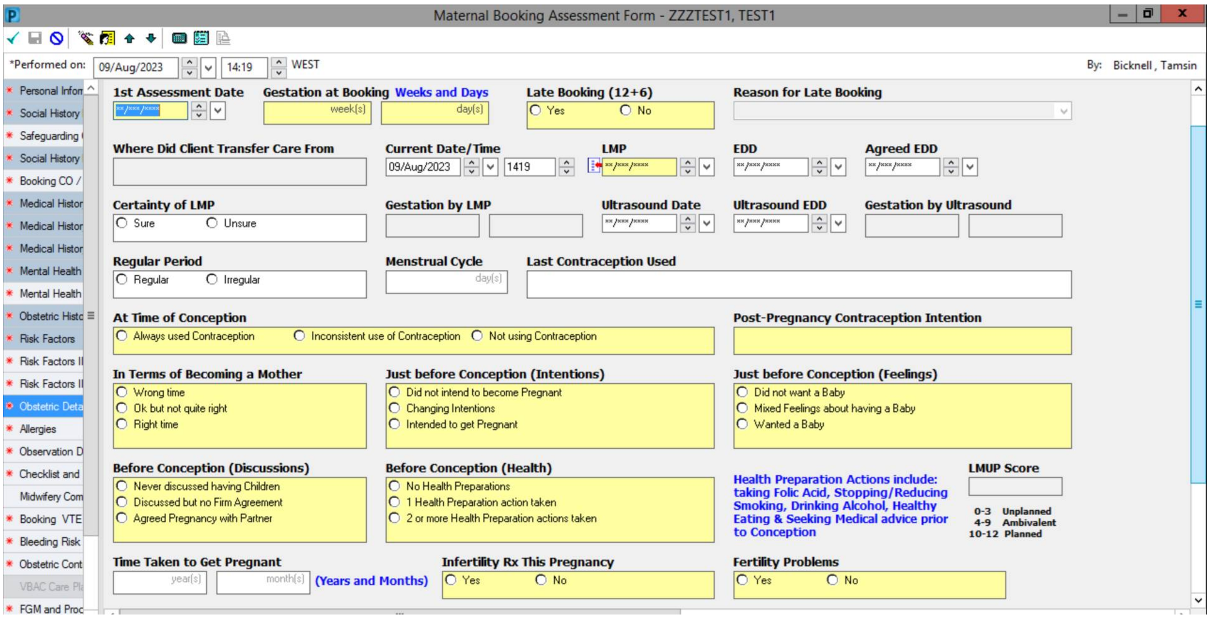

Maternal Booking Assessment Form - ZZZTEST1, TEST1

*Performed on: 09/Aug/2023 14:19 WEST — By: Bicknell , Tamsin

Personal Infor
Social History
Safeguarding
Social History
Booking CO /
Medical Histor
Medical Histor
Medical Histor
Mental Health
Mental Health
Obstetric Histo
Risk Factors
Risk Factors II
Risk Factors II
Obstetric Deta
Allergies
Observation D
Checklist and
Midwifery Com
Booking VTE
Bleeding Risk
Obstetric Cont
VBAC Care Pl
FGM and Proc

1st Assessment Date — **/***/****

Gestation at Booking Weeks and Days — week(s) day(s)

Late Booking (12+6) — ○ Yes ○ No

Reason for Late Booking

Where Did Client Transfer Care From

Current Date/Time — 09/Aug/2023 1419

LMP — **/***/****

EDD — **/***/****

Agreed EDD — **/***/****

Certainty of LMP — ○ Sure ○ Unsure

Gestation by LMP

Ultrasound Date — **/***/****

Ultrasound EDD — **/***/****

Gestation by Ultrasound

Regular Period — ○ Regular ○ Irregular

Menstrual Cycle — day(s)

Last Contraception Used

At Time of Conception — ○ Always used Contraception ○ Inconsistent use of Contraception ○ Not using Contraception

Post-Pregnancy Contraception Intention

In Terms of Becoming a Mother
- ○ Wrong time
- ○ Ok but not quite right
- ○ Right time

Just before Conception (Intentions)
- ○ Did not intend to become Pregnant
- ○ Changing Intentions
- ○ Intended to get Pregnant

Just before Conception (Feelings)
- ○ Did not want a Baby
- ○ Mixed Feelings about having a Baby
- ○ Wanted a Baby

Before Conception (Discussions)
- ○ Never discussed having Children
- ○ Discussed but no Firm Agreement
- ○ Agreed Pregnancy with Partner

Before Conception (Health)
- ○ No Health Preparations
- ○ 1 Health Preparation action taken
- ○ 2 or more Health Preparation actions taken

Health Preparation Actions include: taking Folic Acid, Stopping/Reducing Smoking, Drinking Alcohol, Healthy Eating & Seeking Medical advice prior to Conception

LMUP Score

0-3 Unplanned
4-9 Ambivalent
10-12 Planned

Time Taken to Get Pregnant — year(s) month(s) (Years and Months)

Infertility Rx This Pregnancy — ○ Yes ○ No

Fertility Problems — ○ Yes ○ No

Supporting Information 3 – Midwives Focus Groups Topic Guide

Start of Focus group

- Introductions

- Recap what this research for and answer any questions, collect consent forms and sign in sheet for vouchers

    - Want to get their opinions of the introduction and use of the circumstances of pregnancy questions – what went well, how it could be improved, to find out how they are using the questions, what the barriers are and how these could be addressed.

- Focus group: Approximately 45 mins - 1 hour, £20 voucher as a thank you at end.

- Discuss confidentiality

- Other:
    - Recording
    - Ok to stop or take a break at any time.
    - Set some ground rules.
    - Mobile on silent if possible.
    - Let us know if you want to speak about anything privately at the end

- Any questions?

- Begin recording

General

1. Quick round of introductions with role (clinic / community)

2. Let me start by asking, prior to the introduction of the circumstances of pregnancy questions, how would you assess whether a pregnancy was planned or unplanned, or was this not something that you would routinely do?
   Prompt: Why did you do this? What did you do with this information? Some of the women in our interviews have said that they were asked whether the pregnancy was planned or unplanned – is this something that you do as a lead in to the circumstances of pregnancy questions, or are you asking this as a way of paraphrasing? Did you ask this before the circumstances of pregnancy were added?

3. Now, thinking about your understanding of the circumstances of pregnancy questions, what do you think these questions are trying to achieve? When should you ask them?
   Prompt: what is it measuring? How does it work? Who/when to ask?

About the introduction of the circumstances of pregnancy questions

4. I'd like to talk a bit about the introduction of these questions. How were you first made aware of the introduction of the circumstances of pregnancy questions in the antenatal booking workflow? *How could this have been improved?*

5. What training, if any, have you received about the circumstances of pregnancy questions? If there was training, what was useful and what could have been improved? *Prompt: method, timing, amount, approach*

6. How did you find starting to use the circumstances of pregnancy questions in booking appointments?
Prompt: time, ease, anything else?

Experience of using (or not) the circumstances of pregnancy questions (aim for a free flowing discussion here)

7. OK, great. Now I'd like to move on to talk about your experience. What are your experiences of completing the circumstances of pregnancy questions during booking appointments?
    How do you ask the questions? Full questions or paraphrase / use previous answers?
    Do you find it easy or difficult?
    When during the appointment?
    Always the same way or adapt?
    Are you always able to (fully) complete the circumstances of pregnancy questions?
        If no, please could you tell me about some of these experiences?
    How do you feel whilst completing the circumstances of pregnancy questions? How do you think women feel when being asked? comfortable/uncomfortable with any of the questions asked?

8. What do you think about the fact that so far, in the 13 interviews we have done, while some women wondered why they were being asked, none said that they thought the questions were unacceptable and most could see the value, particularly for unplanned pregnancies?

9. Our interviews and previous FGDs suggest that everyone would prefer a little blurb to introduce the questions. We have developed a few options and would like your opinions on these:
    a. Very general: *The booking appointment is designed for us to get to know you and help us plan your care together. There are a lot of questions, some are very personal or might seem unexpected, but we ask everyone the same questions. We are not here to judge, and have seen it all before, we just want to understand more about you and your pregnancy so that we can provide better care.*
    b. More specific: *The next questions ask about some of the circumstances around your pregnancy and give us an idea of how planned or unplanned a pregnancy may be. We ask everyone these, even though it might not always seem relevant,*

and we are not judging you - it helps us to understand more about you and your pregnancy so that we can provide better care.

c. Most specific: *The next questions ask about some of the circumstances around your pregnancy and give us an idea of how planned or unplanned a pregnancy may be. We ask everyone these, even though it might not always seem relevant, and we are not judging you - it helps us to understand more about you and your pregnancy so that we can provide better care. We know that women and birthing people with unplanned pregnancies may require extra support, so we are keen to identify any extra needs.*

10. We know that there have been some issues with the last question – things that women did before pregnancy. Can you tell us a bit more about how you complete it at the moment and how could this be improved?

11. How could use of the circumstances of pregnancy questions be improved? For example, other ways that women could answer the questions…
Prompt: explore different ways of implementation here - woman self-completes on paper in the waiting room, use of epic app and how this would link to booking...

Impact

12. How has asking the circumstances of pregnancy questions impacted on the booking appointment?
Prompt: too many things to discuss, need longer appointments, uncertain what to do with result, better structure to cover something that you already do or need to know

13. How do you use the results of the circumstances of pregnancy questions?
Prompt: do you use the score or the discussion around the answers?

14. If, when using the circumstances of pregnancy questions, you have identified an unplanned pregnancy what happened next? *Prompt: please could you tell me more about this experience/s - how did this change the woman's care, what services or support are offered? are there other services or support that you think would be beneficial to women with an unplanned pregnancy during pregnancy or beyond?*

15. How do you think using the circumstances of pregnancy questions affects maternity care?
Prompt: Can you tell me why you think that it affects/does not affect care?

16. What problems, if any, have you come up against since you started to use the circumstances of pregnancy questions?
Prompt: practical, organisation, difficult questions from women, presence of partners

17. How do you think COVID affected the roll out of use of the circumstances of pregnancy questions?

Was it easier, more difficult or the same to ask on the phone? Were you more or less likely to ask it, or the same? How did you ask when doing booking over the phone (timing as well as reading v paraphrasing)

Wrap up

18. Any other comments or thoughts about the circumstances of pregnancy questions or anything else anyone would like to add?

End of focus group

- Thank participants. Ask if any questions?
- Stop recording.
- Check if anyone would like to speak privately
- Tell participant how they will receive their voucher.
- Reflexivity: Write up relevant observational notes from the discussion

General probing/follow up suggestions.

'Could you say some more about that…?'; 'What do you mean by that... ?'

Supporting Information 4 – Women's Interviews Topic Guide

Start of interview

- Introductions
- Discuss confidentiality
- Interview: Approximately 45 mins - 1 hour, £20 voucher as a thank you at the end.
- Discuss what this research is for, answer any questions.
- Other:
  - Mode of recording
  - Ok to stop or take a break at any time.
  - You don't have to answer questions you don't want to answer.
  - Mobile on silent if possible.
  - Let us know if you want to speak about anything privately at the end, after we have stopped the recording - this will not be recorded and will not be used
- Any questions?
- Complete consent form / confirm have received it in advance
- If okay to record, **begin recording now**.

Introduction
As we discussed, the aim of this interview is to hear your thoughts about some questions that were recently introduced to the booking appointment (the first antenatal appointment you attend). These questions ask about the circumstances surrounding your pregnancy, such as whether you were using contraception or whether you had done anything in preparation for pregnancy. We would like to know your experience, thoughts and feelings about answering these questions, and whether there is any way we could improve how they are asked or how midwives use what you tell them.

General

1. First of all, it would be really helpful if you could tell me a little bit about you and your pregnancy - whatever you feel comfortable telling me.
   Check how their booking appt was conducted - in person/ zoom etc.

2. [Based on what they say] So it sounds like your pregnancy was fairly planned / unplanned OR if they haven't mentioned: would you describe your pregnancy as more planned / unplanned?

3. Do you think it is important for healthcare professionals to know whether a pregnancy is planned or unplanned? May ask LMUP here if we don't know it.
   Prompt: why do you think it is important/unimportant? Benefit/disadvantages. What do you think healthcare professionals do with the result?

Circumstances of Pregnancy questions

4. What do you recall about being asked questions around the circumstances of your pregnancy?

   How were the questions asked - were they read out to you?
   How did you feel about being asked these questions? Did you find them easy or difficult to understand? Was there anything that concerned or upset you? Surprised? offended? didn't stand out?
   Did you feel comfortable talking to the midwife about the circumstances of your pregnancy?
   Did the midwife give you any explanation of why the questions were being asked? If so, what did they say? If not, did you think you needed some information or did it make sense in the context of your appointment?
   If you can remember, where in the appointment were you asked these questions (beginning / middle / end)?
   Did the midwife tell you your 'score' and what this meant? If not, would you have liked to know?
   Did the midwife have any further discussions with you about whether your pregnancy was planned or unplanned?
   Was anyone with you at the booking appt? did this affect how you felt about answering the questions?

5. Were your thoughts about being asked these questions about the circumstances of your pregnancy mainly positive or negative? What were your thoughts? (prompt: benefits, concerns, disadvantages)

6. Is there anything we could do to improve the way these questions are asked?
   Prompts if needed: For example, ask in a different way, at a different time (earlier / later), have an option to complete them yourself rather than be asked by the midwife, or do you think it is ok how it is?

Impact

7. Do you think that the midwife used this information about the circumstances of your pregnancy in your care plan?
   Why/why not? Do you feel that the midwives followed-up on what you had shared? Could you tell me about the services or support offered as a result of your LMUP result?

8. Are there any other services or support that would be beneficial to you during pregnancy? Or anything else that you would have liked the midwife to do to support you, outside of clinical care, during pregnancy? Or GP/other health professionals?

Wrap up

Thank you so much for your time, it's been really interesting to hear your experience. Before we finish:

9. Do you have any other comments or thoughts about the questions about the circumstances of your pregnancy?

10. Is there anything else I haven't asked that you feel is important to tell me?

11. Check that during the interview you have gained information on age, marital status, ethnicity, gravida.

End of interview

- Thank participant. Ask if any questions?
- **Stop recording**.
- Ask if there is anything that they would like to discuss privately
- Tell participant how they will receive their voucher.
- Ask if they would like to receive a copy of the transcript
- Reflexivity: Write up relevant observational notes from the interview.

General probing/follow up suggestions

'Could you say some more about that…?'; 'What do you mean by that... ?' 'Please tell me more about…'

Supporting information 5 - LMUP Psychometrics

UCLH

At UCLH the Cronbach's alpha for the LMUP was 0.78, demonstrating good reliability. All item-rest correlations were greater than 0.2 and principal components analysis (PCA) confirmed a one factor model (Eigenvalue 3.18). Further details are shown in the table below.

Item	n	Item-rest correlation	Component 1 loadings
**LMUP Question 1**	978	0.35	0.30
**LMUP Question 2**	977	0.66	0.45
**LMUP Question 3**	969	0.71	0.49
**LMUP Question 4**	959	0.65	0.45
**LMUP Question 5**	955	0.68	0.46
**LMUP Question 6**	964	0.35	0.27
**Cronbach's Alpha**	**0.78**		

Cronbach's alpha, item-rest correlations and component 1 loadings for UCLH LMUP data

Hypothesis testing confirmed that unplanned pregnancies were more common in those aged less than 20 years old ($p<0.001$), in unmarried women ($p=0.015$), and in women of parity three or more ($p<0.001$).

HH

At HH the Cronbach's alpha for the LMUP was 0.70, demonstrating acceptable reliability. All item-rest correlations were greater than 0.2 except question 1 (contraception) and principal components analysis (PCA) confirmed a one factor model (Eigenvalue 2.88) but there was a borderline second factor (Eigenvalue 0.99) which mainly represented question 1 (contraception). Investigations into the reason for this are ongoing. Further details are shown in the table below.

Item	n	Item-rest correlation	Component 1 loadings	Component 1 loadings
**LMUP Question 1**	1110	0.08	0.08	0.99
**LMUP Question 2**	1110	0.60	0.48	-0.06
**LMUP Question 3**	1110	0.65	0.49	0.01
**LMUP Question 4**	1110	0.55	0.45	-0.02
**LMUP Question 5**	1110	0.60	0.48	-0.02
**LMUP Question 6**	1110	0.34	0.30	-0.11
**Cronbach's Alpha**	**0.70**			

Cronbach's alpha, item-rest correlations and component 1 loadings for HH LMUP data

Hypothesis testing confirmed that unplanned pregnancies were more common in those aged less than 20 years old ($p<0.001$) and in women of parity three or more ($p<0.001$). Relationship status was not analyses at HH as we only had information on whether the partner detail field had been populated, not whether there was an ongoing relationship.

STH

At STH the Cronbach's alpha for the LMUP was 0.80, demonstrating good reliability. All item-rest correlations were greater than 0.2 and principal components analysis (PCA) confirmed a one factor model (Eigenvalue 3.36). Further details are shown in the table below.

**Item**	**n**	**Item-rest correlation**	**Component 1 loadings**
**LMUP Question 1**	654	0.36	0.27
**LMUP Question 2**	653	0.63	0.44
**LMUP Question 3**	654	0.76	0.49
**LMUP Question 4**	652	0.60	0.42
**LMUP Question 5**	640	0.72	0.47
**LMUP Question 6**	644	0.44	0.31
**Cronbach's Alpha**	**0.78**		

Cronbach's alpha, item-rest correlations and component 1 loadings for STH LMUP data

Hypothesis testing confirmed that unplanned pregnancies were more common in those aged less than 20 years old ($p<0.001$), who were not in a relationship with the biological father ($p<0.001$) and in women of parity three or more ($p<0.001$).

COREQ (COnsolidated criteria for REporting Qualitative research) Checklist

A checklist of items that should be included in reports of qualitative research. You must report the page number in your manuscript where you consider each of the items listed in this checklist. If you have not included this information, either revise your manuscript accordingly before submitting or note N/A.

Topic	Item No.	Guide Questions/Description	Reported on Page No.
**Domain 1: Research team and reflexivity**			
Personal characteristics			
Interviewer/facilitator	1	Which author/s conducted the interview or focus group?	8
Credentials	2	What were the researcher's credentials? E.g. PhD, MD	8
Occupation	3	What was their occupation at the time of the study?	8
Gender	4	Was the researcher male or female?	8
Experience and training	5	What experience or training did the researcher have?	8
Relationship with participants			
Relationship established	6	Was a relationship established prior to study commencement?	8
Participant knowledge of the interviewer	7	What did the participants know about the researcher? e.g. personal goals, reasons for doing the research	8
Interviewer characteristics	8	What characteristics were reported about the inter viewer/facilitator? e.g. Bias, assumptions, reasons and interests in the research topic	8
**Domain 2: Study design**			
Theoretical framework			
Methodological orientation and Theory	9	What methodological orientation was stated to underpin the study? e.g. grounded theory, discourse analysis, ethnography, phenomenology, content analysis	7
Participant selection			
Sampling	10	How were participants selected? e.g. purposive, convenience, consecutive, snowball	6/7
Method of approach	11	How were participants approached? e.g. face-to-face, telephone, mail, email	6/7
Sample size	12	How many participants were in the study?	8-10
Non-participation	13	How many people refused to participate or dropped out? Reasons?	8-11
Setting			
Setting of data collection	14	Where was the data collected? e.g. home, clinic, workplace	7
Presence of non-participants	15	Was anyone else present besides the participants and researchers?	9
Description of sample	16	What are the important characteristics of the sample? e.g. demographic data, date	8-12
Data collection			
Interview guide	17	Were questions, prompts, guides provided by the authors? Was it pilot tested?	7,8
Repeat interviews	18	Were repeat inter views carried out? If yes, how many?	n/a
Audio/visual recording	19	Did the research use audio or visual recording to collect the data?	7
Field notes	20	Were field notes made during and/or after the inter view or focus group?	7
Duration	21	What was the duration of the inter views or focus group?	11
Data saturation	22	Was data saturation discussed?	information p[illegible]
Transcripts returned	23	Were transcripts returned to participants for comment and/or	7

Topic	Item No.	Guide Questions/Description	Reported on Page No.
		correction?	
**Domain 3: analysis and findings**			
Data analysis			
Number of data coders	24	How many data coders coded the data?	7,
Description of the coding tree	25	Did authors provide a description of the coding tree?	13, figure 1
Derivation of themes	26	Were themes identified in advance or derived from the data?	7
Software	27	What software, if applicable, was used to manage the data?	7
Participant checking	28	Did participants provide feedback on the findings?	7, 16
Reporting			
Quotations presented	29	Were participant quotations presented to illustrate the themes/findings? Was each quotation identified? e.g. participant number	table 4
Data and findings consistent	30	Was there consistency between the data presented and the findings?	12-20
Clarity of major themes	31	Were major themes clearly presented in the findings?	12-20 and Figu
Clarity of minor themes	32	Is there a description of diverse cases or discussion of minor themes?	12-20

Developed from: Tong A, Sainsbury P, Craig J. Consolidated criteria for reporting qualitative research (COREQ): a 32-item checklist for interviews and focus groups. *International Journal for Quality in Health Care*. 2007. Volume 19, Number 6: pp. 349 – 357

Once you have completed this checklist, please save a copy and upload it as part of your submission. DO NOT include this checklist as part of the main manuscript document. It must be uploaded as a separate file.

© 2024 Hall J.A. et al.
